# Enhancing emotional intelligence through sleep therapy in high-stress professionals: a case-control study on efficacy and predictive factors

**DOI:** 10.3389/fpsyt.2025.1637904

**Published:** 2025-11-13

**Authors:** Yujia Zhai, Weiqiang Ji, Yugui Li, Qingfeng Du

**Affiliations:** 1 Endocrinology Department, The Seventh Affiliated Hospital, Southern Medical University, Foshan, Guangdong, China; 2 Health Management Division, Hospital of Integrated Traditional Chinese and Western Medicine, Southern Medical University, Guangzhou, Guangdong, China; 3 Traditional Chinese Medicine Department, The Third People’s Hospital of Bengbu, Bengbu, Anhui, China; 4 Centre of General Practice, The Seventh Affiliated Hospital, Southern Medical University, Foshan, Guangdong, China; 5 Guangdong Provincial Key Laboratory of Chinese Medicine Pharmaceutics, School of Traditional Chinese Medicine, Southern Medical University, Guangzhou, Guangdong, China; 6 Guangdong Basic Research Center of Excellence for Integrated Traditional and Western Medicine for Qingzhi Diseases, Hospital of Integrated Traditional Chinese and Western Medicine, Southern Medical University, Guangzhou, Guangdong, China

**Keywords:** sleep therapy, emotional intelligence, sleep disorders, occupational health, adherence, psychological assessment

## Abstract

**Background:**

Sleep disturbances can significantly impair emotional intelligence (EI), particularly among professionals in high-stress occupations. This study evaluated the effectiveness of sleep therapy (ST) in enhancing EI and identified key predictors of therapeutic success.

**Methods:**

We conducted a retrospective analysis of 471 adults from high-stress professions who completed a 12-week intervention at Bengbu Third People’s Hospital. Among them, 214 received psychoeducational treatment and 257 underwent sleep therapy. A case-control study was performed on the 257 sleep therapy patients. Pre- and post-treatment assessments measured EI components, sleep quality, psychological status, and physical health. Patients were stratified into high and low EI groups based on post-treatment EIS scores. Univariate and multivariate logistic regression analyses identified predictors of EIS improvement.

**Results:**

Following the 12-week intervention, the ST group showed significantly greater improvement in total EIS score (60.87 *vs*. 58.39, P<0.001) compared to a psychoeducational therapy group. Multivariate analysis identified ST intervention (P<0.001) and higher adherence (P = 0.002) as significant protective factors for EI, whereas increasing age was a risk factor (P = 0.001). Compared to the low EI group, the high EI group demonstrated significantly better baseline adherence (70.7% *vs*. 52.6% full adherence, P = 0.010), greater sleep improvement (ΔPSQI: 3.2 *vs*. 1.9, P<0.001), lower anxiety (SAS: 46.6 *vs*. 49.2, P = 0.006), and higher self-esteem (SES: 31.8 *vs*. 30.4, P<0.001).

**Conclusion:**

Sleep therapy effectively enhances EI in high-stress professionals suffering from sleep disorders. Treatment efficacy is strongly influenced by adherence, baseline psychological and physical health status, and sleep quality improvements.

## 1 Introduction

The complex relationship between sleep, cognitive functioning, and emotional well-being has garnered increasing attention, particularly within high-stress occupational environments characterized by elevated psychological demands (1). In high-pressure occupational settings, job demands often exceeded individuals’ coping resources, creating a persistent imbalance (2). These environments were characterized by high workloads, critical decision-making responsibilities, emotional exhaustion, and unpredictability. High-pressure professionals referred to those who worked long-term in such environments and typically needed exceptional emotional resilience and cognitive sharpness to handle these challenges (3). Professions such as those in healthcare, law enforcement, aviation, and emergency response require exceptional emotional resilience and cognitive acuity to navigate high-stakes scenarios high-stakes environments (4). An essential element supporting these attributes is emotional intelligence (EI), which encompasses the capacity to recognize, understand, and manage not only one’s emotions but also the emotions of others (5). This construct is critical for maintaining interpersonal interactions and decision-making processes under stress (6).

EI was a core resource for effective stress management. Individuals with high EI could more accurately assess stressors and use emotion regulation strategies to alleviate anxiety and frustration, thereby maintaining the availability of cognitive resources (7). This was particularly important for making clear decisions in critical situations. In professions that heavily relied on teamwork, such as medical teams and rescue groups, accurately interpreting nonverbal emotional cues from colleagues and service recipients, such as patients or the public, formed the foundation for building trust, effective communication, and collaborative problem-solving (8). Additionally, self-emotion management helped prevent impulsive behavior and emotional exhaustion under stress, which was crucial for maintaining professional integrity and personal mental health (9). Therefore, this construct played a vital role in sustaining effective interpersonal interactions and decision-making processes under pressure.

Ironically, individuals in such professions often predispose individuals to poor sleep patterns due to irregular shifts, prolonged working hours, and chronic exposure to occupational stressors (10). The concept of “poor sleep patterns” was complex and primarily caused by occupational characteristics, manifesting as disruptions in the sleep-wake cycle (11). Specifically, this included irregular sleep timing, such as daytime sleep due to shift work, insufficient sleep duration due to long working hours or being on call, and low sleep efficiency, which refers to difficulty falling or staying asleep because of psychophysiological arousal or external disturbances. For example, a firefighter returning from a late-night emergency call might find it difficult to fall asleep quickly afterward, despite physical exhaustion, because of heightened alertness following the incident. Sleep deprivation and poor sleep quality are well-documented to impair cognitive functions and emotional regulation, thereby undermining the EI essential to optimal job performance (12). Research data further highlighted the prevalence and severity of this issue. International studies showed that the prevalence of insomnia symptoms among healthcare professionals ranged from 30% to 70%, significantly higher than in the general population (13). Domestic regional studies also pointed to similar findings. For example, a survey of police officers in a certain province found that over 50% of respondents experienced sleep quality issues as assessed by the Pittsburgh Sleep Quality Index (PSQI) (14). The main contributing factors included workload, shift work, and psychological stress. Despite the prevalence of sleep-related disturbances in high-stress professions, therapeutic interventions targeting sleep quality, specifically their impact on EI, are not extensively studied (15). Addressing this gap, sleep therapy (ST) emerges as a promising intervention due to its focus on optimizing sleep duration and efficiency through the regulation of sleep-wake cycles and the consolidation of restful periods.

ST was a behavioral intervention based on sleep timing and limitation principles. Its core involved systematically adjusting time in bed to better match an individual’s actual sleep needs, thereby consolidating sleep and improving sleep efficiency (16). Participants recorded sleep diaries to calculate the average weekly sleep efficiency (sleep time divided by time in bed multiplied by 100%). Based on preset efficiency thresholds, bedtime was dynamically adjusted: if the efficiency met the standard, bedtime was slightly advanced; if it was too high, it remained unchanged; if it was too low, bedtime was delayed. This method aimed to rebuild a stable and efficient sleep-wake cycle through behavioral shaping. ST’s potential benefits stem from its ability to instigate a more structured sleep pattern, thereby alleviating the cognitive and emotional deficits associated with sleep deprivation (17). The restoration of sleep has been linked to enhance the prefrontal cortex’s functioning, a brain region integral to emotional regulation and decision-making (18). Furthermore, improved sleep quality has been linked to better mood regulation, reduced anxiety and depression symptoms, and more adaptive social functioning, all of which are components closely tied to EI.

Previous studies have largely concentrated on the physiological consequences of inadequate sleep, while relatively few studies have addressed its psychological and emotional impacts within occupational contexts (19). This knowledge gap calls for the need for targeted investigations into the efficacy of sleep interventions like ST within these fields, alongside an exploration of factors influencing their efficacy.

This study aims to investigate the impact of ST on EI among individuals engaged in high-stress occupations. It evaluates the therapy’s effectiveness and the various factors that may affect outcomes. Furthermore, we aim to identify baseline characteristics and behavioral factors, such as adherence to the therapy and initial psychological well-being, that might modulate the therapeutic benefits of ST.

## 2 Materials and methods

### 2.1 Case selection

We initially conducted a retrospective cohort study to evaluate the effects of different treatment methods on patients with sleep disorders. A total of 471 patients with sleep disorders who were treated at Bengbu Third People’s Hospital between December 2022 and December 2023 were included in the study. Among them, 214 patients received psychoeducational therapy, while 257 patients received standard sleep therapy. Subsequently, we performed a case-control study using the data from the 257 patients who received sleep therapy.

Inclusion criteria comprised of the following: participants must be aged between 18 and 65 years. Participants were drawn from high-stress professions, with this study focusing on healthcare, aviation, emergency services, and law enforcement. Both day-shift and night-shift workers were included. Healthcare professionals referred to frontline clinical staff who provided direct patient care, including physicians, nurses, and emergency center healthcare workers. Aviation personnel referred to active pilots engaged in commercial flights. Emergency service workers included first responders involved in pre-hospital emergency care and disaster response, such as firefighters and ambulance paramedics. Law enforcement officers were defined as frontline police personnel responsible for patrol and law enforcement duties. These professions were chosen due to their shared characteristics: high workloads, critical decision-making responsibilities, frequent exposure to emotional or traumatic events, and the need for irregular or extended working hours, including shift work. These factors collectively created a typical high-risk environment for sleep disorders and challenges in emotional intelligence. Participants needed to meet the criteria of the Occupational Stress Index (OSI), which required a score of 50 or higher on the Work Stressor Inventory (WSI) (20). Meet the diagnostic criteria for sleep disorders (21). Participants needed to demonstrate normal cognitive function, assessed by a score of 26 or higher on the Montreal Cognitive Assessment Basic Level (MoCA-BL), sufficient for completing questionnaire surveys and understanding intervention guidance (22).

Exclusion criteria included individuals suffering from serious mental illnesses (e.g., schizophrenia, and bipolar disorder) who are currently receiving treatment or experiencing instability; those with severe physical health conditions, such as heart, liver, or kidney diseases; pregnant or lactating women; and patients unable to commit to full participation in the study.

This study received approval from the Ethics Committee of the Third People’s Hospital of Bengbu’s. Written informed consent was obtained from all participants. All patient data used in this study were de-identified and there was no potential risk or adverse impact on participants during the study.

### 2.2 Intervening method

In the cohort study, all 471 patients received a 12-week intervention. Among them, patients receiving psychoeducational therapy (n=214) attended biweekly psychoeducational sessions led by professional psychological counselors. The course content included stress management techniques, cognitive-behavioral therapy (CBT), and emotion regulation strategies.

For patients receiving standard sleep therapy (ST), the treatment protocol involved developing reasonable bedtimes and wake-up times based on each participant’s work schedule. Participants were instructed to maintain a sleep diary, documenting their bedtime, wake-up time, and actual sleep duration, and to provide this feedback to the researchers. Sleep efficiency was calculated weekly based on the recorded data using the formula: (actual sleep time/total bed rest time) × 100%. If sleep efficiency reached 75% or above, participants were permitted to go to bed 15 minutes earlier; if it ranged from 80% to 90%, they maintained their current bedtime; and if it fell below 80%, bedtime was delayed by 15 minutes. The intervention lasted for a total duration of 12 weeks.

For the case-control study, the grouping criteria were established to compare the emotional intelligence levels of patients before and after a 12-week sleep therapy (ST) intervention. Based on their emotional intelligence scores following the intervention, participants were categorized into two groups: a high emotional intelligence group consisting of 181 individuals, each with a total score exceeding 60 points on the Emotional Intelligence Scale (EIS), and a low emotional intelligence group comprising 76 individuals, each with a total score of 60 points or lower. In this study, we selected a total EIS score of 60 as the clinical cutoff for group classification. This is because the maximum score on the EIS is 80, and a score of 60 represents 75% of the total possible score. In behavioral science, a 75% score is often used to determine whether an individual has reached a “good” or “proficient” level in a particular psychological construct. Therefore, this threshold helps us identify individuals who have shown significant improvement in emotional intelligence and notable functional enhancement in clinical practice.

### 2.3 Data collection and outcome measurement

We systematically collected demographic information from the patient records, which encompassed general patient data and emotional intelligence levels before and following treatment. After 12 weeks of treatment, patients were categorized based on their EI scores, allowing for an analysis of their demographic information as well as their psychological and physical health status.

#### 2.3.1 Compliance assessment

The researchers assessed patient compliance using sleep diaries, categorizing it into three levels: complete compliance, partial compliance, and non-compliance, with corresponding scores of 9-10, 4-8, and 1-3, respectively. A higher score indicated greater compliance with the sleep therapy protocol.

#### 2.3.2 Pittsburgh sleep quality index

The Pittsburgh sleep quality index (PSQI) is an appropriate tool for assessing sleep quality in individuals with sleep disorders and mental health conditions, as well as in the general population. The PSQI evaluates participants’ sleep quality over the previous month and includes 19 self-assessment items along with 5 additional self-evaluation items. However, only the 18 items that contribute to scoring are considered in this study (see attached questionnaire for further details). These 18 items are organized into 7 components, each scored on a scale from 0 to 3. The cumulative score across all components yields the total PSQI score, which ranges from 0 to 21, with higher scores indicating poorer sleep quality. The scale demonstrated a Cronbach’s alpha coefficient of 0.71, indicating acceptable reliability (23).

#### 2.3.3 Emotional Intelligence Scale

The level of EI was assessed using the Emotional Intelligence Scale (EIS), which comprises 4 dimensions and a total of 16 items: self-emotion assessment (items 1-4), self-emotion management (items 5-8), self-emotion utilization (items 9-12), and evaluation of others’ emotions (items 13-16). The scale employs a 5-point Likert rating system, where responses range from “strongly disagree” to “strongly agree”, corresponding to scores of 1 to 5, respectively. The total EIS is calculated by summing the individual item scores, with higher scores indicating greater emotional intelligence. The EIS demonstrated strong reliability, with an overall Cronbach’s alpha coefficient of 0.885, while the Cronbach’s alpha coefficients for the 4 dimensions ranged from 0.826 to 0.904 (24).

#### 2.3.4 Psychological assessment

The Self-Rating Depression Scale (SDS) was used to assess negative emotions experienced by patients during treatment, with scores ranging from 0 to 100. Higher scores indicate a greater degree of negative emotions, and the scale exhibited a Cronbach’s alpha coefficient of 0.92 (25). In addition, the Self-Rating Anxiety Scale (SAS) serves as a straightforward clinical tool for evaluating subjective anxiety symptoms in patients. It uses a 4-point rating system that primarily measures the frequency of symptom occurrence. The ratings are defined as follows: “1” for no or very little time, “2” for sometimes, “3” for most of the time, and “4” for most or all the time. Among the 20 items, 15 are phrased negatively and scored by the previously mentioned scale, while 5 items (5, 9, 13, 17, and 19) are worded positively and scored in reverse (4-1). The SAS has a cutoff score of 50, with scores between 50–59 indicating mild anxiety, 60–69 indicating moderate anxiety, and 70 or above indicating severe anxiety, and it demonstrated a Cronbach’s alpha coefficient of 0.897 (26).

Furthermore, the Self-Esteem Scale (SES) assesses an individual’s overall perception of self-worth and self-acceptance, consisting of 10 items. Items 3, 5, 8, 9, and 10 are reverse-scored using a 4-point Likert scale, from “strongly disagree” (1 point) to “strongly agree” (4 points), yielding a total score range of 10 to 40 points. Higher scores reflect elevated levels of self-esteem, with thresholds of low self-esteem defined as below 25 points, moderate self-esteem between 26 and 32 points, and high self-esteem above 33 points. The SES exhibited a Cronbach’s alpha coefficient of 0.86 (27).

#### 2.3.5 Health status assessment

The Physical Component Summary (PCS) is a key component of the SF-12 scale, designed to evaluate an individual’s physical health status. It encompasses several dimensions, including bodily function, bodily roles, bodily pain, general health, vitality, and social functioning. The PCS score is calculated using standardized algorithms that integrate these dimensions into a single score, reflecting overall physical health, with a range from 0 to 100. Higher scores indicate better physical health, and the PCS displayed a Cronbach’s coefficient of 0.743 (28). Additionally, the EQ-5D scale (EuroQol five-dimension three-level scale), developed by the EuroQol Group, is a widely recognized tool for assessing health-related quality of life. This scale consists of five dimensions (mobility, self-care, daily activities, pain/discomfort, and anxiety/depression) where respondents select a level of response for each dimension. The scores are aggregated to create a comprehensive health status description, with a total score of 100 points. A higher score signifies better health status. The EQ-5D-3L demonstrated good internal consistency, achieving an overall Cronbach’s alpha of 0.75 (29).

### 2.4 Statistical methods

The measurement data are presented as mean ± standard deviation ( $\bar{\text{x}}$ ± s). Categorical data are reported in terms of frequency and percentage. Continuous variables between the two groups were compared using unpaired t-tests. Univariate and multivariate logistic regression analyses were performed to calculate the odds ratios (OR) and 95% confidence intervals (CI) for each parameter treated as a continuous variable. Statistical significance was set at *P* < 0.05. All statistical analyses were carried out using SPSS software version 22 (SPSS Inc., Chicago, IL, USA) and R software package version 3.0.2 (Free Software Foundation, Inc., Boston, MA, USA).

## 3 Results

### 3.1 Demographic and baseline characteristics of the study population

In the study population, demographic and baseline characteristics were compared between participants undergoing psychoeducational therapy and those receiving sleep therapy (**Table 1**). No significant differences were observed across a range of parameters (all P > 0.05). These findings indicate that the two groups were well-matched at baseline, enhancing the comparability of the intervention effects between psychoeducational therapy and sleep therapy.

**Table 1:** Demographic and baseline characteristics of the study population.

Parameters	Psychoeducational therapy (n=214)	Sleep therapy (n=257)	t/χ^2^	P Value
Age (years)	39.82 ± 9.38	40.60 ± 9.22	0.917	0.359
Body Mass Index (kg/m²)	22.56 ± 2.43	22.48 ± 2.37	0.358	0.721
Gender (male/female)	125 (58.41%)/89 (41.59%)	140 (54.47%)/117 (45.53%)	0.735	0.391
Education Level [n/(%)]			0.266	0.876
- Primary School	8 (3.74%)	8 (3.11%)		
-Secondary School	33 (15.42%)	43 (16.73%)		
- College	173 (80.84%)	206 (80.16%)		
Marital Status [n/(%)]			0.719	0.698
- Married	148 (69.16%)	169 (65.76%)		
- Single	51 (23.83%)	66 (25.68%)		
- Divorced	15 (7.01%)	22 (8.56%)		
Occupation Type			0.901	0.825
- Healthcare Workers	63 (29.44%)	77 (29.96%)		
- Police Officers	44 (20.56%)	46 (17.90%)		
- Firefighters	48 (22.43%)	55 (21.40%)		
- Pilots	59 (27.57%)	79 (30.74%)		
Average Monthly Income (RMB)			1.152	0.765
- < 3000	15 (7.01%)	23 (8.95%)		
-3000~5999	68 (31.78%)	87 (33.85%)		
-6000~9000	83 (38.79%)	90 (35.02%)		
- > 9000	48 (22.43%)	57 (22.18%)		
Smoking [n (%)]	58 (27.10%)	64 (24.90%)	0.294	0.587
Drinking [n (%)]	39 (18.22%)	43 (16.73%)	0.181	0.671
Diabetes [n (%)]	18 (8.41%)	20 (7.78%)	0.062	0.803
Hypertension [n (%)]	33 (15.42%)	41 (15.95%)	0.025	0.874
Adherence [n (%)]			0.770	0.680
- Full Adherence	147 (68.69%)	168 (65.37%)		
- Partial Adherence	51 (23.83%)	65 (25.29%)		
- Non-Adherence	16 (7.48%)	24 (9.34%)		
History of Sleep Disorders [n (%)]	16 (7.48%)	19 (7.39%)	0.001	0.973
PSQI score (points)	9.79 ± 2.35	9.81 ± 2.43	0.096	0.923

### 3.2 Comparison of emotional intelligence levels of patients before and after treatment

Before treatment, there were no significant differences between the two groups in terms of Self-Emotion Assessment, Self-Emotion Management, Self-Emotion Utilization, Other-Emotion Assessment, and Total Score (all P > 0.05), indicating similar baseline emotional statuses and capabilities (**Table 2**). After treatment, significant differences were observed across several measures. The sleep therapy group demonstrated significantly higher scores than the psychoeducational therapy group in Self-Emotion Assessment (P = 0.013), Self-Emotion Management (P = 0.004), Self-Emotion Utilization (P = 0.009), and Other-Emotion Assessment (P = 0.039). Additionally, a significant difference was found in the Total Score between the two groups post-treatment (P < 0.001), favoring the sleep therapy group. These results suggest that compared to psychoeducational therapy, sleep therapy may be more effective in enhancing participants’ abilities in various aspects of emotional assessment and management following the intervention period.

**Table 2:** Comparison of emotional intelligence levels of patients before and after treatment.

Parameters	Psychoeducational therapy (n=214)	Sleep therapy (n=257)	t/χ^2^	P Value
Before treatment				
Self-Emotion Assessment (points)	12.19 ± 3.25	12.21 ± 3.13	0.042	0.966
Self-Emotion Management (points)	13.82 ± 3.71	13.93 ± 3.18	0.351	0.726
Self-Emotion Utilization (points)	13.81 ± 3.34	13.76 ± 2.61	0.164	0.870
Other-Emotion Assessment (points)	13.64 ± 3.27	13.58 ± 2.95	0.235	0.814
Total Score	53.46 ± 6.81	53.48 ± 6.15	0.029	0.977
After treatment				
Self-Emotion Assessment (points)	14.03 ± 2.86	14.68 ± 2.79	2.492	0.013
Self-Emotion Management (points)	15.12 ± 2.34	15.8 ± 2.71	2.916	0.004
Self-Emotion Utilization (points)	14.33 ± 2.73	14.97 ± 2.60	2.628	0.009
Other-Emotion Assessment (points)	14.91 ± 2.89	15.42 ± 2.54	2.067	0.039
Total Score	58.39 ± 5.72	60.87 ± 5.38	4.851	< 0.001

### 3.3 Multifactorial analysis of factors affecting emotional intelligence

In the multifactorial analysis examining factors affecting emotional intelligence, several significant predictors were identified (**Table 3**). Age was found to be a significant factor, with each one-year increase associated with a decrease in emotional intelligence (P = 0.003), indicating age as a potential risk factor. Gender differences did not reach statistical significance (P = 0.816). Treatment type emerged as a significant predictor, with sleep therapy showing a positive association compared to psychoeducational therapy (P < 0.001). This indicates that receiving sleep therapy is a protective factor for enhancing emotional intelligence. Adherence also significantly predicted higher emotional intelligence (P = 0.002), further acting as a protective factor; higher adherence levels were linked to improved outcomes. A history of sleep disorders did not show a significant association with emotional intelligence (P = 0.830). The baseline EIS total score was another significant predictor, with each one-point increase at baseline being positively associated with emotional intelligence post-intervention (P = 0.012), thus serving as a protective factor. Overall, the analysis highlights sleep therapy and adherence as key protective factors for enhancing emotional intelligence, while age appears to pose a slight risk. Baseline emotional intelligence also plays a protective role, underscoring the importance of early assessment and intervention.

**Table 3:** Multifactorial analysis of factors affecting emotional intelligence.

Parameters	Coefficient	Std error	Wald stat	P	OR (95% CI)
Age (per 1-year increase)	-0.033	0.010	-3.238	0.001	0.967 (0.948-0.987)
Gender (male *vs*. female)	-0.208	0.217	-0.961	0.816	0.536 (1.242-0.340)
Treatment (Sleep therapy *vs*. Psychoeducational therapy)	0.720	0.194	3.708	<0.001	2.055 (1.404-3.007)
Adherence	0.613	0.198	3.093	0.002	1.846 (1.252-2.722)
History of Sleep Disorders (yes *vs*. no)	-0.187	0.125	-1.492	0.830	0.649 (1.060-0.136)
Baseline EIS total score (per 1-score increased)	0.050	0.020	2.518	0.012	1.051 (1.011-1.093)

### 3.4 General information analysis of patients grouped by therapeutic effect on emotional intelligence

High-efficacy participants (n=181) demonstrated superior baseline adherence (70.7% *vs* 52.6% full adherence, P = 0.010) and greater PSQI improvement (ΔPSQI=3.2 *vs* 1.9, P<0.001) compared to low responders (n=76). No demographic differences were observed between groups (**Table 4**).

**Table 4:** General information analysis of patients grouped by therapeutic effect on emotional intelligence.

Parameters	High group (n=181)	Low group (n=76)	t/χ^2^	P Value
Age (years)	40.21 ± 9.17	41.53 ± 9.34	1.046	0.296
Body Mass Index (kg/m²)	25.32 ± 3.15	25.87 ± 3.31	1.244	0.214
Gender (male/female)	98 (54.14%)/83 (45.86%)	42 (55.26%)/34 (44.74%)	0.027	0.869
Education Level [n/(%)]			0.299	0.861
- Primary School	5 (2.76%)	3 (3.95%)		
- Secondary School	31 (17.13%)	12 (15.79%)		
- College	145 (80.11%)	61 (80.26%)		
Marital Status [n/(%)]			0.615	0.735
- Married	121 (66.85%)	48 (63.16%)		
- Single	46 (25.41%)	20 (26.32%)		
- Divorced	14 (7.73%)	8 (10.53%)		
Occupation Type			0.084	0.994
- Healthcare Workers	55 (30.39%)	22 (28.95%)		
- Police Officers	32 (17.68%)	14 (18.42%)		
- Firefighters	39 (21.55%)	16 (21.05%)		
- Pilots	55 (30.39%)	24 (31.58%)		
Average Monthly Income (RMB)			0.168	0.983
- < 3000	16 (8.84%)	7 (9.21%)		
-3000~5999	62 (34.25%)	25 (32.89%)		
-6000~9000	64 (35.36%)	26 (34.21%)		
- > 9000	39 (21.55%)	18 (23.68%)		
Smoking (yes/no)	44 (24.31%)/137 (75.69%)	20 (26.32%)/56 (73.68%)	0.115	0.734
Drinking (yes/no)	31 (17.13%)/150 (82.87%)	12 (15.79%)/64 (84.21%)	0.069	0.793
Diabetes [n/(%)]	11 (6.08%)/170 (93.92%)	9 (11.84%)/67 (88.16%)	2.479	0.115
Hypertension [n/(%)]	25 (13.81%)/156 (86.19%)	16 (21.05%)/60 (78.95%)	2.093	0.148
Baseline adherence [n (%)]			9.174	0.010
- Full Adherence	128 (70.72%)	40 (52.63%)		
- Partial Adherence	41 (22.65%)	24 (31.58%)		
- Non-Adherence	12 (6.63%)	12 (15.79%)		
History of Sleep Disorders	10 (5.52%)	9 (11.84%)	3.120	0.077
PSQI score (points)	6.73 ± 2.14	8.16 ± 2.34	4.741	< 0.001
EIS Total Score (points)	71.26 ± 8.33	46.87 ± 11.42	16.836	< 0.001

PSQI, Pittsburgh sleep quality index; EIS, Emotional Intelligence Scale

### 3.5 Psychological assessment of two groups of patients upon enrollment

**Figure 1:**
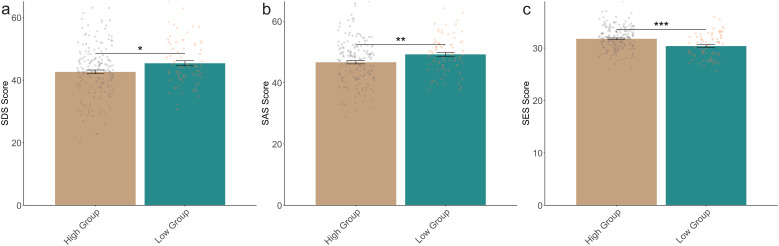
Psychological assessment of two groups of patients upon enrollment. **(A)**: SDS Score; **(B)**: SAS Score; **(C)**: SES Score. SDS, Self-rating Depression Scale; SAS, Self-rating anxiety scale; SES, Self-Esteem Scale. *: P<0.05; **: P<0.01; ***: P<0.001.

Baseline psychological profiles differentiated groups: high-efficacy participants had 6.5% lower anxiety (SAS 46.6 *vs* 49.2, P = 0.006) and 4.6% higher self-esteem (SES 31.8 *vs* 30.4, P<0.001) (**Figure 1**).

### 3.6 Comparison of health status between two groups of patients upon enrollment

**Figure 2:**
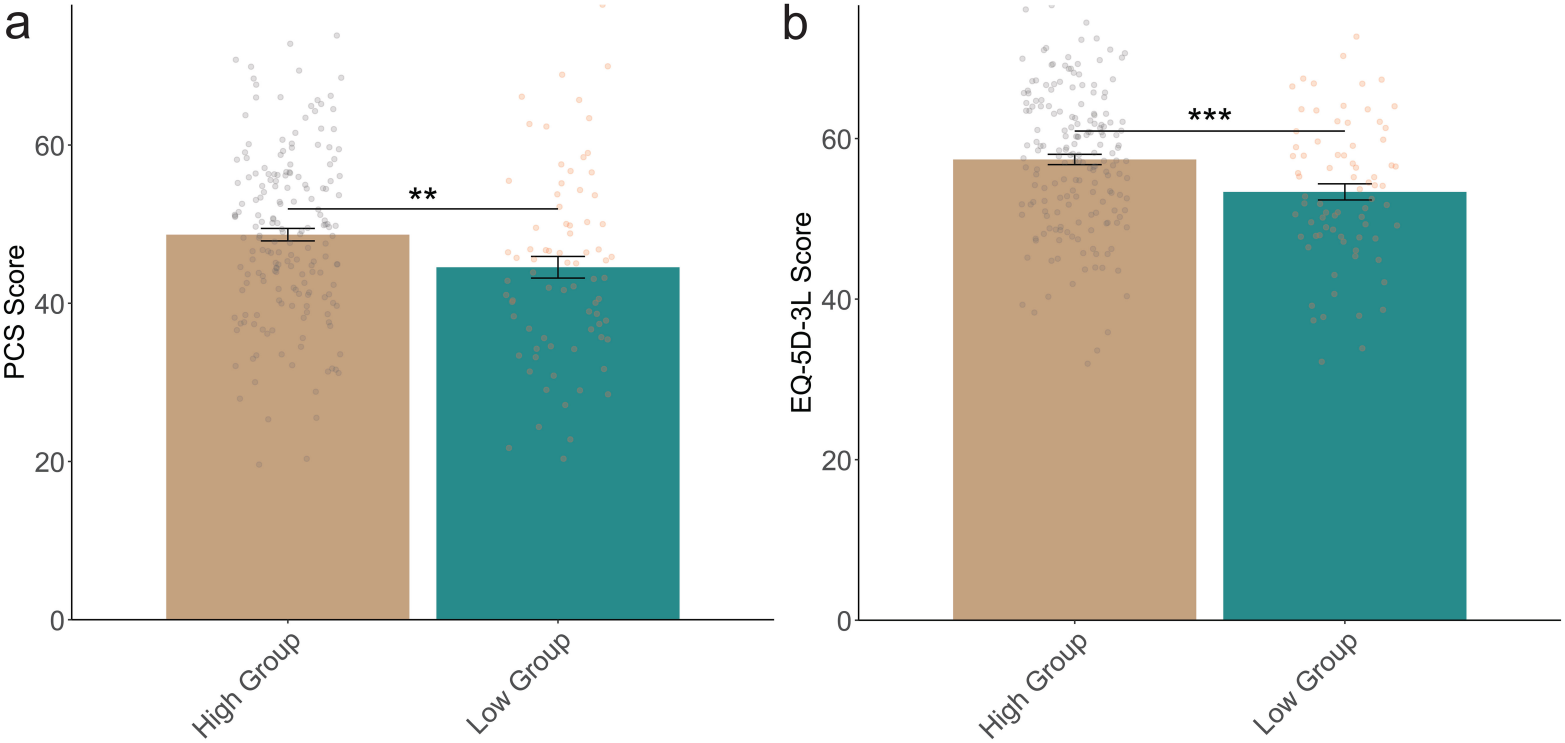
Comparison of health status between two groups of patients upon enrollment. **(A)**: PCS Score; **(B)**: EQ-5D-3L Score. PCS, Physical Component Summary; EQ-5D-3L, EuroQol five-dimension three-level questionnaire. **: P<0.01; ***: P<0.001.

Upon enrollment, the health status assessment revealed significant differences in physical health and overall quality of life between the high and low effect groups. The high group reported a higher Physical Component Summary (PCS) score, indicating better physical health, with a mean of 48.67 ± 10.61 compared to 44.55 ± 11.93 in the low group (*t=*2.736, *P=*0.007) (**Figure 2**). Additionally, the EuroQol five-dimension three-level (EQ-5D-3L) score, which reflects overall health-related quality of life, was significantly higher in the high group at 57.39 ± 8.63, compared to 53.36 ± 8.77 in the low group (*t=*3.408, *P* < 0.001). These results suggest that better baseline physical health and quality of life are associated with more significant improvements in emotional intelligence following sleep therapy.

### 3.7 Correlation analysis between poor treatment effect of sleep therapy and various parameters

Correlation analysis demonstrated several significant associations between parameters and the efficacy of sleep therapy on emotional intelligence (**Table 5**). Adherence to therapy displayed a negative correlation with poor treatment effect (rho=-0.184, *P=*0.003), suggesting that higher adherence is linked to better outcomes. A positive correlation was observed between the Pittsburgh Sleep Quality Index (PSQI) score and poor treatment effect (rho=0.299, *P* < 0.001), indicating that poorer sleep quality is associated with reduced efficacy. Both the Self-rating Depression Scale (SDS) and Self-rating Anxiety Scale (SAS) scores showed positive correlations with poor treatment outcomes (rho=0.137, *P=*0.028 and rho=0.167, *P=*0.007, respectively), highlighting the negative impact of depressive and anxiety symptoms. Conversely, the Self-Esteem Scale (SES) score was negatively correlated with poor treatment effect (rho=-0.265, *P* < 0.001), implying that higher self-esteem predicts better outcomes. Similarly, the Physical Component Summary (PCS) and EQ-5D-3L scores were negatively correlated with poor treatment effect (rho=-0.176, *P=*0.005 and rho=-0.204, *P* < 0.001, respectively), suggesting that better physical health and quality of life enhance the therapy’s efficacy. These findings underscore the importance of adherence, baseline psychological well-being, and overall health in improving the therapeutic outcomes of sleep therapy.

**Table 5:** Correlation analysis between poor treatment effect of sleep therapy and various parameters.

Parameters	rho	P Value
Adherence	-0.184	0.003
PSQI score	0.299	< 0.001
SDS Score	0.137	0.028
SAS Score	0.167	0.007
SES Score	-0.265	< 0.001
PCS Score	-0.176	0.005
EQ-5D-3L Score	-0.204	< 0.001

SDS, Self-rating Depression Scale; SAS, Self-rating anxiety scale; SES, Self-Esteem Scale; PCS, Physical Component Summary; EQ-5D scale (Euro Qol five-dimension three-level scale).

### 3.8 Logistic regression analysis between poor treatment effect of sleep therapy and various parameters

The single-factor logistic regression analysis identified several significant predictors of the poor treatment effect of sleep therapy (**Table 6**). Higher adherence was associated with reduced odds of poor treatment outcomes (Coefficien*t=*-0.595, *P=*0.003, O*R=0.552*, 95% CI: 0.371–0.817). Conversely, increased PSQI scores were linked to higher odds of poor outcomes (Coefficien*t=*0.301, *P* < 0.001, O*R=*1.352, 95% CI: 1.186–1.558), as were higher SDS (*P=*0.012) and SAS scores (*P=*0.007), with ORs of 1.046 and 1.057, respectively. Notably, higher SES and PCS scores were associated with better treatment outcomes (SES Coefficien*t=*-0.289, *P* < 0.001, O*R=*0.749; PCS Coefficien*t=*-0.034, *P=*0.008, O*R=*0.966), while the EQ-5D-3L also indicated a protective effect (Coefficien*t=*-0.053, *P=*0.001, O*R=*0.949).

**Table 6:** Single factor logistic regression analysis between poor treatment effect of sleep therapy and various parameters.

	Coefficient	Std error	Wald	P value	OR	CI lower	CI upper
Adherence	-0.595	0.201	-2.967	0.003	0.552	0.371	0.817
PSQI score	0.301	0.069	4.341	< 0.001	1.352	1.186	1.558
SDS Score	0.045	0.018	2.522	0.012	1.046	1.011	1.085
SAS Score	0.055	0.021	2.681	0.007	1.057	1.016	1.102
SES Score	-0.289	0.068	4.286	< 0.001	0.749	0.653	0.851
PCS Score	-0.034	0.013	2.670	0.008	0.966	0.942	0.991
EQ-5D-3L Score	-0.053	0.016	3.271	0.001	0.949	0.918	0.978

Multivariate logistic regression corroborated these findings, with adherence (Coefficien*t=*-0.872, *P* =<.001, O*R=*0.418) and PSQI scores (Coefficien*t* = 0.257, *P* < 0.001, O*R=*1.293) remaining significant predictors after adjusting for covariates (**Table 7**). The SES score continued to be a strong indicator of better outcomes (Coefficien*t=*-0.317, *P=* <.001, O*R=*0.728), and the EQ-5D-3L score demonstrated a similar protective trend (Coefficien*t* = -0.058, *P=*0.003, O*R=*0.944). Notably, PCS score showed borderline significance (Coefficien*t* = -0.043, *P=*0.006, O*R=*0.958) in influencing treatment efficacy.

**Table 7:** Multivariate logistic regression analysis between poor treatment effect of sleep therapy and various parameters.

	Coefficient	Std error	Wald_Stat	P Value	OR	OR_CI lower	OR_CI upper
Adherence	-0.872	0.238	-3.669	<.001	0.418	0.262	0.666
PSQI score	0.257	0.077	3.340	<.001	1.293	1.112	1.503
SDS Score	0.032	0.021	1.507	0.132	1.033	0.990	1.077
SAS Score	0.056	0.024	2.294	0.022	1.057	1.008	1.109
SES Score	-0.317	0.079	-4.029	<.001	0.728	0.624	0.850
PCS Score	-0.043	0.015	-2.761	0.006	0.958	0.929	0.988
EQ-5D-3L Score	-0.058	0.019	-3.001	0.003	0.944	0.909	0.980

## 4 Discussion

In the emerging field of occupational health, particularly within high-stress professions, the intersection of sleep quality and EI presents a fascinating and critical area of study for investigation. Our study, which explored the impact of ST on EI among individuals in such high-pressure roles, revealed significant post-intervention improvements in emotional intelligence. Furthermore, it identified a range of interrelated factors contributing to the therapy’s overall effectiveness.

The observed benefits of ST are consistent with a study during the chronic stress of the COVID-19 pandemic, which showed that high-quality sleep provides a foundation for adaptive cognitive-emotional regulation strategies by maintaining good executive function, helping individuals resist depression and anxiety, thus directly supporting the notion that improved sleep facilitates emotional regulation (30). The mechanism behind this relationship is likely related to the restorative functions of sleep, which play a critical role in modulating brain regions involved in emotional processing such as the prefrontal cortex and the amygdala (31). By promoting better quality sleep, ST may help optimize the functioning of these neural substrates, thereby enhancing core dimensions of EI, including self-awareness, emotional control, and interpersonal empathy (32).

A particularly notable outcome of our findings was the significant enhancement in self-emotion assessment, regulation, and utilization, highlighting the critical role of sleep in nurturing intrapersonal emotional competencies. This improvement was comparable to that achieved through mindfulness interventions in teacher populations, although our intervention period was shorter (33). Sleep insufficiency is known to impair emotional regulation and increase emotional reactivity, primarily due to altered activity in neuroregulatory circuits. ST, by alleviating these sleep-related disruptions, seems to facilitate an increased ability to understand and modulate one’s emotions (34). This improvement could be essential for individuals in high-stress occupations where emotional resilience is of primary relevance for sustaining professional performance and psychological stability.

Moreover, the specific increase in ‘evaluation of others’ emotions’ following ST may reflect enhanced interpersonal EI. Sleep impacted social interactions, previous studies demonstrated that sleep deprivation not only heightened perceptions of social threats but also directly impaired various aspects of empathy, such as empathic concern, perspective-taking ability, and empathic sensitivity (35). The self-regulatory benefits of ST might reduce such biases, thereby improving the ability to accurately perceive and interpret the emotional states of others. This finding complemented the results of studies using the “mind-reading” task, which demonstrated that sleep deprivation impaired individuals’ ability to recognize others’ emotional states (36). Our intervention, on the other hand, provided evidence from a positive perspective, showing that sleep restoration enhanced this ability. This capacity is particularly essential in professions such as healthcare and law enforcement, where human interactions are frequent and intense (37).

Our study further highlights adherence as a major factor influencing the success of ST. This finding aligns with established behavioral intervention literature, where consistent engagement with prescribed strategies often dictates therapeutic success. We observed that approximately one-third of the participants did not fully adhere to the intervention, a phenomenon worthy of further exploration. The relatively low rate of full adherence in the study could be attributed to multiple factors. First, the inherent characteristics of the participants’ professions, such as unpredictable work schedules, frequent night shifts, and emergency tasks, conflicted with the structured and regular sleep-wake routines required by ST (38). Second, sleep restriction, a core component of ST, often led to increased subjective sleepiness and fatigue during the initial stages, which might reduce participants’ motivation and perceived efficacy, thereby hindering their continued adherence (39). Additionally, individual factors such as lower baseline self-esteem and higher anxiety levels, known predictors of poorer outcomes, likely contributed to reduced self-efficacy and persistence in adhering to the behavioral protocol (40). It highlights the necessity for supporting strategies to enhance adherence among participants, such as behavioral coaching or the incorporation of feedback mechanisms in therapy design. Importantly, adherence as a predictor of therapeutic success is consistent with broader behavioral intervention frameworks, where fidelity to regimen is often a determinant of efficacy.

Interestingly, baseline sleep quality, as indicated by the PSQI, is a robust predictor of treatment outcomes. Individuals with poorer initial sleep may experience greater barriers to benefiting from ST. This is an important consideration for clinicians, as it emphasizes the need for personalized treatment plans that might involve complementary interventions. Future clinical practice could consider combining ST with other evidence-supported interventions (41). For instance, cognitive behavioral therapy for insomnia helped address persistent sleep-related thoughts and behaviors. Mindfulness-based interventions or acceptance and commitment therapy improved awareness and acceptance of nighttime awakenings and negative emotions (42). Sleep hygiene education laid the groundwork for behavioral changes (43). Additionally, methods such as light therapy for shift workers and music therapy could serve as beneficial supplements, depending on individual needs and the source of their sleep issues.

The moderating role of baseline mental health, particularly anxiety, was another key finding. Elevated anxiety levels were associated with diminished therapeutic gains, echoing existing literature which highlights the bidirectional interplay between psychological distress and sleep dysfunction. Emotional disorders can impair sleep and hinder the benefits of sleep interventions. Thus, embedding comprehensive mental health support within sleep-focused treatments may amplify improvements in EI.

On the other hand, higher self-esteem emerged as a predictor of positive therapeutic outcomes. This relationship underlines the role self-perception plays in health behavior and intervention responsiveness. Individuals with higher self-esteem may present better coping strategies and a positive outlook that enhance their engagement with and responses to therapy (44). This finding aligned with studies in the field of chronic disease management, which consistently reported that high self-esteem was a predictor of better self-management behaviors among patients (45). This suggests that initial assessments of self-esteem may inform the tailoring of interventions, perhaps by incorporating elements that build self-efficacy alongside traditional sleep therapies.

Finally, our study points to the important contributions of physical health and health-related quality of life as factors influencing the efficacy of ST in enhancing EI. This finding may be explained by the bi-directional relationship between physical health and sleep quality, where poor physical health exacerbates sleep problems, potentially blunting the effects of sleep treatments (46). The protective effect of superior physical health on ST outcomes highlights the benefits of adopting a holistic approach that considers physical health maintenance as part of an integrated intervention strategy, promoting overall well-being and maximizing therapeutic outcomes.

Despite these valuable insights, our study has some limitations. First, our study design relied on self-reported data collected through questionnaires, which are inherently subject to various biases, such as recall bias and social desirability bias. In addition, the study was conducted within a specific cohort with certain demographic and occupational characteristics, potentially limiting the generalizability of our findings to other populations or occupational settings. Furthermore, potential confounding factors such as individual resilience traits, lifestyle variables (e.g., diet, exercise), and concurrent stress exposures were not controlled, possibly influencing both sleep quality and EI. Finally, the absence of a randomized control group limits the ability to draw causal inferences from our findings. Future studies should aim to address these limitations by employing prospective, randomized controlled designs and incorporating a broader range of influencing factors.

Future research could explore comprehensive treatment methods that combine sleep optimization strategies with support measures to enhance psychological resilience and physical health. This holistic approach includes personalized sleep optimization plans, such as CBT-I based on individual baseline sleep quality assessments or improvements in sleep hygiene; psychological resilience training, which employs mindfulness training, emotion regulation techniques, and stress management courses to help individuals better cope with stress; healthy lifestyle guidance, offering nutritional counseling, exercise plans, and advice on smoking cessation and alcohol moderation to promote overall well-being; and the development of social support systems, leveraging support from family, friends, and professional counseling to assist individuals in managing various life challenges.

## 5 Conclusion

This study found that sleep therapy effectively improved emotional intelligence among high-stress professionals. The effectiveness depended on treatment adherence, baseline sleep quality, and mental health. These findings had clear implications for clinical practice, future interventions should combine sleep optimization with psychological support to better enhance occupational well-being and job performance among professionals.

## Data Availability

The original contributions presented in the study are included in the article/supplementary material. Further inquiries can be directed to the corresponding authors.
